# Health and safety implications of recruitment payments in migrant construction workers

**DOI:** 10.1093/occmed/kqu018

**Published:** 2014-03-25

**Authors:** H. A. Hassan, J. Houdmont

**Affiliations:** Division of Psychiatry and Applied Psychology, School of Medicine, University of Nottingham, Nottingham NG8 1BB, UK.

**Keywords:** Accidents, economic stress, health, labour recruiters, migrant construction workers, safety.

## Abstract

**Background:**

The Middle East construction sector is heavily reliant on a migrant workforce that predominantly originates from South Asia. It is common practice for migrant construction workers to pay a local labour recruiter the equivalent of one or more years’ prospective overseas salary to secure employment, work and travel permits and transportation. The occupational health and safety implications of these financial arrangements remain unexplored.

**Aims:**

To examine associations between payment to a labour recruiter, perceived general health and worksite accidents among migrant construction workers in the Middle East.

**Methods:**

A questionnaire was completed by a convenience sample of predominantly Indian migrant construction workers drawn from a large construction project. The relationship between payment and risk of poor health and workplace accidents was assessed using multivariate logistic regression models (crude and adjusted for socio-demographic and occupational factors).

**Results:**

There were 651 participants. The majority (58%) of migrant construction workers had paid a labour recruiter and ~40% had experienced a worksite accident. Between 3% (labourers) and 9% (foremen) perceived their health to be poor. Labourers and skilled workers who had paid a labour recruiter were significantly more likely to have experienced a worksite accident in the previous 12 months. Skilled workers, but not labourers and foremen, who had paid a labour recruiter were at increased risk of poor health.

**Conclusions:**

The mechanisms linking labour recruiter payments to adverse safety and health outcomes warrant investigation with a view to developing interventions to erode these links.

## Introduction

The migrant workforce in the Middle East amounted to an estimated 13 million workers in 2005 [1]. Approximately half of male migrant workers in the region are employed in the construction sector [2], and the United Arab Emirates (UAE) alone was estimated to employ some 700000 migrant construction workers in 2006 [3]. Migrant construction workers, predominantly drawn from South Asian countries, often secure employment, work and travel visas, and transportation to the Middle East via the services of locally owned labour recruitment companies to which a sum of money equivalent to one or more years of their prospective overseas salary is paid [4]. In addition to an official payment, it is common for additional illegal fees to be paid. One study of Indian migrant returnees found that recruiters’ fees were typically over 6000% more than the legally permitted maximum [3]. Workers who lack the capital required to pay a labour recruiter usually take out one or more loans, often at a high rate of interest [3]. It is not uncommon for migration debts to be repaid over a period of several years [5], a timeframe of two to three times longer than commonly expected prior to migration [3]. This disparity between expectation and reality for repayment periods may partly be explained by misleading information given by labour recruiters concerning migrant construction workers’ overseas earning potential [4]. The economic conditions of migrant construction workers who secure employment via a local recruiter are particularly harsh in comparison to those whose labour is hired directly by an employer. Buckley found that ‘respondents employed by these agencies tended to have no fixed salary, no direct relationship with the contractors running their work site and no access to severance pay or subsidized flights home. Most importantly they also tended not even to have a short-term guarantee of work; depending on the availability of subcontracts on other work sites these workers faced the perpetual risk of enduring indeterminate periods of time in which they had no work and no pay cheque’ [3]. There is evidence to suggest that the prevalence of occupational accidents and injuries among migrant workers in the Middle East is particularly high. Accidental injuries were the second most frequently reported cause of death in the UAE in 2004 [6], and the leading cause in the largest Emirate, Abu Dhabi, in 2007 [7]. Examination of occupational injury hospitalizations in a large city within the UAE revealed 614 admissions over a 25-month period, just 4% of which involved Emirati nationals. The primary cause of injury was falls (51%) followed by falling objects (15%) [8]. Even less is known about the health of migrant construction workers in the Middle East. One survey of Bangladeshi migrant workers found that 60% of males reported that their health had improved and 60% reported having put on weight following migration to the UAE. Improved nutrition was cited as the primary factor accounting for these changes. Few workers reported that their health had worsened after migration [2].

Transactional stress theory [9] conceptualizes a process linking (i) antecedent factors, namely poorly designed, managed and organized aspects of work (‘stressors’) with (ii) cognitive perceptual processes that give rise to the emotional experience of stress and (iii) correlates, or outcomes, of that experience. Within this framework, the range of possible stressors may include economic stressors, defined as ‘aspects of economic life that are potential stressors for employees and their families and consist of both objective and subjective components reflecting the employment and income dimensions of the worker-earner role’ [10]. Support for this per spective can be found in the scientific literature that illustrates relations between economic stressors and job attitudes and performance, health, intention to leave and suicide [11]. According to transactional stress theory, it is reasonable to expect that the presence of an economic stressor in the form of payment having been made to a labour recruiter might be associated with occupational health and safety impairment. Taken together, theory and evidence allow for the hypothesis that immigrant workers who have paid a labour recruiter to secure employment in the Middle East construction sector are more likely than those who have not made such a payment to experience poor health and workplace accidents. In exploring associations between recruiter payments and occupational outcomes, this exploratory study sought to examine a practice that has been neglected in terms of its possible implications for workers’ health and safety.

## Methods

The study took place in 2012 within a large oil and gas facility construction project in the Middle East. Ethical approval was granted by the research ethics committee of the Institute of Work, Health and Organizations at the University of Nottingham, and the research followed the British Psychological Society’s (2009) code of ethics and conduct [12]. With the support of the main contractor, a series of focus groups involving 50 migrant construction workers was undertaken with the aim of identifying aspects of work experienced as stressful. The requirement to fulfil labour recruiter repayment obligations was identified as the single most noteworthy stressor. In response, a questionnaire was developed to examine labour recruiter payment arrangements, health status and involvement in worksite accidents. The questionnaire was provided in English (the construction project’s official operational language), Hindi and Urdu. Translations were made in accordance with the Brislin guidelines [13]. After piloting, the questionnaire and an envelope in which to seal it when completed was administered by safety representatives to a convenience sample of migrant construction workers. Responses were anonymous and returned to safety representatives for forwarding to the first author.

All variables were measured using a single self-report questionnaire. In addition to socio-demographic and occupational characteristics (age, gender, nationality, education, number of dependents, tenure), data were collected on labour recruiter payment, with a single question, ‘Did you pay a labour recruiter in order to secure your current job?’, with responses in a dichotomous ‘yes/no’ format; on workplace accidents, with a single question, ‘In the last year how many accidents have you had at work?’, with responses dichotomized into 0 (not involved in an accident) and 1 (involved in one or more accidents); and on self-rated health, which, following Leineweber *et al.* [14], was measured by the question ‘How would you rate your general state of health?’, with responses on a five-point scale from 1 (very good) to 5 (very poor), which were dichotomized into ‘good/average’ (1–3) and ‘poor’ (4 or 5). A single-item measure of health was applied in order to ensure brevity in the questionnaire and encourage a strong response rate. This measure has been shown to predict morbidity and mortality in a variety of epidemiological investigations [15,16]. Data contributed by labourers, skilled workers and foremen were analysed separately owing to differences between these three groups in terms of demographic composition and type of work undertaken. The relationship between labour recruiter payments, poor general health and involvement in workplace accidents was measured by odds ratios (ORs) and 95% confidence intervals (CIs) using multivariate logistic regression models. For each worker group, the likelihood of having had an occupational accident in the last 12 months and of poor health was established among those who had paid a labour recruiter relative to those who had not made such payment. To control for the effects of socio-demographic and occupational covariates, we also used a model adjusted for age, education, number of dependents and tenure in the organization. Data were screened prior to analysis for the accuracy of scores, missing data, outliers and violations of the assumptions of regression analysis. All statistical analyses were undertaken using IBM SPPS Statistics Version 21 [17].

## Results

Questionnaires were distributed to 1000 migrant construction workers, and 651 completed and useable questionnaires (65% response rate) were returned. Socio-demographic and occupational characteristics of the sample are presented in Table 1. The frequency of labour recruiter payments, poor health and involvement in accidents was different for the three groups of workers (Table 2). Notably, and irrespective of the employee type, the majority of workers had paid a labour recruiter and ~40% had experienced a workplace accident. Between 3% (labourers) and 9% (foremen) reported poor perceived health.

**Table 1.** Respondents’ socio-demographic and occupational characteristics

	Labourers	Skilled workers	Foremen
	*n* (%)	*n* (%)	*n* (%)
Socio-demographic characteristics			
Gender			
Male	188 (100)	337 (100)	126 (100)
Age			
16–20	–	3 (1)	–
21–25	35 (19)	34 (10)	36 (29)
26–30	85 (45)	143 (42)	42 (33)
31–35	33 (18	80 (24)	23 (18)
36–40	18 (10)	45 (13)	6 (5)
41–45	12 (6)	22 (6)	6 (5)
46–50	3 (2)	2 (1)	4 (3)
51+	2 (1)	5 (1)	3 (2)
Not specified	–	3 (1)	6 (5)
Marital status			
Single	53 (28)	91 (27)	55 (44)
Married	131 (70)	240 (71	64 (51)
Divorced	2 (1)	3 (1)	4 (3)
Not specified	2 (1)	3 (1)	3 (2)
Nationality			
Bangladeshi	3 (2)	8 (2)	2 (2)
Egyptian	–	–	1 (1)
Filipino	1 (<1)	1 (<1)	9 (7)
Indian	147 (78)	269 (80)	105 (83)
Jordanian	–	2 (1)	–
Nepali	2 (1)	4 (1)	–
Nigerian	–	1 (<1)	1 (1)
Pakistani	31 (16)	37 (11)	1 (1)
Syrian	–	1 (<1)	2 (2)
Turkish	1 (<1)	–	1 (1)
Vietnamese	3 (2)	11 (3)	3 (2)
Not specified	–	3 (1)	1 (1)
Education (years)			
0–5	17 (9)	13 (4)	2 (2)
6–10	127 (68)	192 (57)	14 (11)
11–15	37 (20)	120 (35)	62 (49)
16–20	7 (4)	8 (2)	41 (32)
Not specified	–	4 (1)	7 (6)
Dependents			
0–4	40 (21)	103 (30)	52 (41)
5–9	98 (52)	180 (53)	54 (43)
10–14	24 (13)	39 (12)	7 (6)
15–19	8 (4)	5 (1)	3 (2)
20+	5 (3)	3 (1)	10 (8)
Not specified	13 (7)	7 (2)	–
Occupational characteristics			
Tenure			
<6 months	45 (24)	38 (11)	24 (19)
6 months–1 year	42 (22)	97 (29)	18 (14)
1–2 years	50 (27)	122 (36)	54 (43)
>2 years	51 (27)	80 (24)	30 (24)

**Table 2.** Frequencies (%) of labour recruiter payments, poor health and involvement in accidents

	Labourers	Skilled workers	Foremen
Labour recruiter payment	59	64	52
Poor health	3	5	9
Accident involvement	41	43	43

Risk of poor health and involvement in an accident associated with labour recruiter payment are shown in Table 3. Labourers who had paid a labour recruiter were more likely to have experienced a workplace accident in the previous 12 months (crude OR). This relationship remained significant when socio-demographic characteristics were controlled for (Model 1) but failed to reach statistical significance when tenure in the organization was controlled for in the regression model (Model 2). Skilled workers who had paid a labour recruiter were more likely to have experienced a workplace accident in the previous 12 months, and this relationship remained significant when controlling for the influence of both socio-demographic and occupational factors. Foremen who had paid a labour recruiter were at no increased risk for accidents. Payment to a labour recruiter was not associated with increased risk of poor health among labourers or foremen. A significantly increased risk was found for skilled workers, but with large CIs associated with both crude and adjusted ORs suggesting that these findings should be considered with extreme caution.

**Table 3.** ORs (95% CIs) of labour recruiter payments and risk of poor health and accident involvement

	Poor health			Accident involvement		
	Crude	Model 1^a^	Model 2^b^	Crude	Model 1^a^	Model 2^b^
Labourers (*n* = 188)	3.57 (0.41–31.20)	3.61 (0.39–33.46)	1.48 (0.68–3.19)	**3.12 (1.66–5.88)**	**3.24 (1.63–6.47)**	1.10 (0.84–1.44)
Skilled workers (*n* = 337)	**10.27 (1.35–78.16)**	**14.35 (1.75–117.81)**	**12.53 (1.49–105.40)**	**2.24 (1.40–3.59)**	**2.36 (1.45–3.85)**	**2.57 (1.55–4.26)**
Foremen (*n* = 126)	0.92 (0.28–3.01)	0.41 (0.07–2.56)	0.35 (0.53–2.36)	0.59 (0.29–1.20)	0.53 (0.24–1.17)	0.52 (0.24–1.16)

Significant findings are in bold.

^a^Controlled for socio-demographic factors (age, education and number of dependents).

^b^Controlled for socio-demographic factors (age, education and number of dependents) plus occupational factors (tenure).

## Discussion

This study found that the majority of migrant construction workers had paid a labour recruiter and ~40% had experienced a workplace accident. Fewer than 1 in 10 respondents perceived their health to be poor. Labourers and skilled workers (but not foremen) who had paid a labour recruiter were more likely to have experienced a workplace accident in the previous 12 months. After controlling for a variety of possible socio-demographic confounders, the ORs remained significant. When job tenure was added to the regression model, only the OR for skilled workers remained significant.

In this study, the association between payment to a labour recruiter and increased risk of reported poor health among skilled workers was statistically significant. However, the extremely broad CI (1.35–78.2) suggests that this finding should be interpreted with caution. The findings concerning health are generally consistent with the limited literature on the health of the migrant workforce in the Middle East, which indicates that health sometimes improves after migration, a situation that has been attributed to, among other things, improved nutrition [2]. Nevertheless, the findings concerning health are perhaps surprising given the poor living conditions of many migrant construction workers in the region. Future studies could usefully examine the objective health status of the construction migrant workforce in order to ascertain whether this is consistent with subjective perceptions and whether health moderates or mediates the relationship between recruiter payment and worksite accidents.

There are multiple possible explanations for the association found in this study between labour recruiter payment and safety. Among these is the plausible explanation that workers perceive the imperative to maximize income generation to be incompatible with safety policies and procedures. This might be so particularly where workers are paid on the basis of productivity or where overtime is available, both of which provide an opportunity for income enhancement. Indeed qualitative research involving interviews with construction workers in Hong Kong has shown that the opportunity to enhance earnings through additional productivity can encourage workers to commit unsafe acts [18]. It is also possible that migrant construction workers in our study who paid a labour recruiter (and were therefore not directly employed by the main contractor) were less familiar with and, by extension, more likely to breach the site’s safety rules and regulations than those who were employed directly, resulting in an elevated accident rate. It is important that future studies seek to identify the mechanisms that link labour recruiter payments with occupational safety. Knowledge of these could aid the development of interventions to erode the links between these phenomena. The literature on the linkage between job insecurity and occupational safety might provide some useful insights because job insecurity is associated with workplace injuries and accidents owing to decreased safety motivation and compliance [19] and workplace safety culture might moderate the job security–safety relationship [20]. It is possible that labour recruitment arrangements generate an inflated accident risk via interactions with immigration policies, societal hierarchies and wage theft that is particularly prevalent in the construction sector and which can cause migrant workers to miss monthly payments to moneylenders back home [21].

The absence of a relationship between labour recruiter payment and risk of accident involvement among foremen in this study might be attributable to, among other possible factors, lower inherent opportunities for accidents in the type of work undertaken by foremen, education or the availability of more equitable financial arrangements for foremen. In order to reduce accident risk among labourers and skilled workers, future studies should attempt to identify the protective factors that apply to foremen in this regard.

This study has a number of limitations. Firstly, it is possible that the safety climate might have had a moderating influence on the relationship between labour recruiter payment and health and safety outcomes that is not accounted for in the findings. Safety climate has been shown to have a moderating influence on the relationship between job insecurity and safety beyond the construction context [20]. In order to test for the possible moderating influence of safety climate, future studies should account for this. Changes in the ORs that resulted from the addition of socio-demographic and occupational characteristics to the regression models suggest that these likewise have an influence on the relationship. There are a number of possible factors that might help to explain this; for example, the greater the number of dependents back home the greater the possible negative implications of failing to send home sufficient money each month and, by extension, the greater the possible temptation to cut corners and breach safety regulations. Future qualitative studies could usefully explore the mechanisms by which these and other socio-demographic and occupational variables influence the relationship between labour recruiter payment and occupational safety. Secondly, the study may have been limited by the selection and quality of measures. Single-item measures were used to ensure questionnaire brevity and maximize the response rate. Given the limitations of such measures, it is possible that use of a multi-item and multi-dimensional health measure might have produced contrasting results. Future studies should use multi-item scales of both physical and psychological health in order to establish whether the current finding results from a Type II error. Thirdly, the cross-sectional research design means that the possibility of common method variance cannot be disregarded. However, several procedural remedies were introduced in order to reduce the possibility of this problem [22]. These included the use of anonymous responses and use of established scales. Future research could usefully establish the predictive effects of labour recruiter payments on health and safety using a longitudinal research design. Fourthly, we measured current health status only, which prevented us from controlling for the possible influence of pre-migration health status on the outcome variables. To address this limitation, future studies ought to measure perceived change in health status and, where possible, measure health pre-migration. Finally, participants in this study were drawn from a single construction site and were self-selecting, limiting the generalizability of the findings to the Middle East construction sector as a whole.

Despite these limitations, the results of this exploratory study indicate that South Asian migrant construction workers, working in the Middle East, are at increased risk of involvement in occupational accidents and poor health if they have made a payment to a labour recruiter. In shining a spotlight on the existence of this risk, this study seeks to stimulate further research on a neglected topic with a view to developing interventions to promote the health and safety of migrant construction workers.

**Key points**

- The majority of migrant construction workers surveyed in the Middle East had paid a labour recruiter in their home country and ~40% had experienced a worksite accident in the last year. However, fewer than 1 in 10 reported poor perceived health.
- Migrant construction workers (labourers and skilled workers) who had paid a labour recruiter were more likely to have experienced one or more occupational accidents. Skilled workers were more likely to report poor health.
- The mechanisms linking labour recruiter payment to safety warrant further investigation with a view to developing interventions designed to erode such links and improve workplace safety.

## Conflicts of interest

None declared.

## References

[1] Suter B Labour Migration in the United Arab Emirates: Field Study on Regular and Irregular Migration in Dubai. Sweden: Malmo University, 2005

[2] Rahman M Bangladeshi migrant workers in the UAE: gender-differentiated patterns of migration experiences. Middle Eastern Stud 2011;47:395–411

[3] Buckley M From Kerala to Dubai and back again: construction migrant workers and the global economic crisis. Geoforum 2012;43:250–259

[4] Human Rights Watch. Building Towers, Cheating Workers: Exploitation of Migrant Construction Workers in the United Arab Emirates. New York: Human Rights Watch, 2006

[5] Rajan S Prakash B Migration and Development Linkages Re-examined in the Context of the Global Economic Crisis. Paper presented at the 3rd Global Forum on Migration and Development in Athens, Greece, November 2–3, 2009

[6] Preventive Medicine Sector. Annual Report 2004. Abu Dhabi, United Arab Emirates: Ministry of Health, 2006

[7] Health Authority of Abu Dhabi. Health statistics 2008. In: Health Authority—Abu Dhabi Annual Report 2008. Abu Dhabi, United Arab Emirates: Emirate of Abu Dhabi, 2008; 110

[8] Barss P Addley K Grivna M Stanculescu C Abu-Zidan F Occupational injury in the United Arab Emirates: epidemiology and prevention. Occup Med (Lond) 2009;59:493–498 19640929 10.1093/occmed/kqp101

[9] Cox T Griffiths A Work-related stress: a theoretical perspective. In: Leka S Houdmont J, eds. Occupational Health Psychology. Oxford: Wiley-Blackwell, 2010;31–56

[10] Probst T Economic stressors. In: Barling J Kelloway K Frone M, eds. Handbook of Work Stress. Thousand Oaks, CA: Sage, 2005;267–297

[11] Sinclair R Sears L Probst T Zajack M A multilevel model of economic stress and employee well-being. In: Houdmont J Leka S, eds. Contemporary Occupational Health Psychology: Global Perspectives on Research and Practice. Vol. 1 Chichester, UK: Wiley-Blackwell, 2010;1–20

[12] British Psychological Society. Code of Ethics and Conduct. London: British Psychological Society, 2009

[13] Brislin R Back-translation for cross-cultural research. J Cross Cult Psychol 1970;1:185–216

[14] Leineweber C Wege N Westerlund H Theorell T Wahrendorf M Siegrist J How valid is a short measure of effort-reward imbalance at work? A replication study from Sweden. Occup Environ Med 2010;67:526–531 20573847 10.1136/oem.2009.050930

[15] Benyamini Y Idler E Community studies reporting association between self-rated health and mortality: additional studies, 1995 to 1999. Res Aging 1999;21:392–401

[16] DeSalvo KB Bloser N Reynolds K He J Muntner P Mortality prediction with a single general self-rated health question. A meta-analysis. J Gen Intern Med 2006;21:267–275 16336622 10.1111/j.1525-1497.2005.00291.x PMC1828094

[17] IBM. SPSS Statistics Version 21. Somers, NY: IBM, 2012

[18] Choudhry R Fang D Why operatives engage in unsafe work behavior: investigating factors on construction sites. Saf Sci 2008;46:566–584

[19] Probst TM Brubaker TL The effects of job insecurity on employee safety outcomes: cross-sectional and longitudinal explorations. J Occup Health Psychol 2001;6:139–159 11326726

[20] Probst TM Safety and insecurity: exploring the moderating effect of organizational safety climate. J Occup Health Psychol 2004;9:3–10 14700454 10.1037/1076-8998.9.1.3

[21] Human Rights Watch. The Island of Happiness: Exploitation of Migrant Workers on Saadiyat Island, Abu Dhabi. New York: Human Rights Watch, 2009

[22] Podsakoff PM MacKenzie SB Lee JY Podsakoff NP Common method biases in behavioral research: a critical review of the literature and recommended remedies. J Appl Psychol 2003;88:879–903 14516251 10.1037/0021-9010.88.5.879

